# Excerpts

**Published:** 1889-09

**Authors:** 


### Excerpts.

For polishing purposes Dr. F. A. Williamson, Fort Scott, Kan., wraps sandpaper on a hardwood mandrel, glueing it firmly, laying a few folds of old rubber dam inside to make it more pliable. A cork, turned to any desired shape, and covered with sandpaper will also be found useful in reaching angles, etc. For applying polishing powder, he cuts wheels from heavy machinery bands of rubber and cloth, also of sole leather; they cost nothing and last a life time.

---

Dr. B. A. Stevens, Hannibal, Mo., treats root canals with peroxide, chloroform or alcohol, hot air, carbolic acid or creosote, in the order named. He then moistens a string of cotton with creosote and passes it to the end of the root, leaving one end out by which to remove it subsequently. On this he presses tightly a pellet of cotton, over this a cone of warm red gutta percha and over a pellet of cotton and sandpaper. The patient is then dismissed from three days to a week. If no trouble ensues, this is all removed and the canal permanently filled. If pus is found he treats with carbolic acid and iodine, and repeats the temporary stopping for a few days longer.

---

Dr. B. F. Luckey, Patterson, N. J., reports a case of slight paralysis of the lower part of the face following the insertion of a dressing of iodoform and creosote, sealed with gutta percha in the pulp canal of a lower right bicuspid.

---

Fees.—Dr. John G. Harper, St. Louis, says "time is money" to the dentist as to any other man. Bills promptly rendered are, as a rule, promptly paid; they should therefore be presented at the completion of a series of sittings. In the case of family patients monthly collections should be made. A deposit should be required from strangers, with payment at the completion of each sitting.

---

Emil Mallinckradt, of the Mallinckradt Chemical Works, St.

Louis, says: "The commercial article of peroxide of hydrogen in general use is a 3 per cent. aqueous solution, or nearly a ten-volume solution, the relation of percentage to volume being 1 to 3.29.

---

Dr. H. Fisher, St. Louis, Mo., says that the sulphuric acid generally used to aid in the preservation of peroxide of hydrogen, may be neutralized by adding baryte water, drop by drop, till turbidity ceases. Thus neutralized it forms a valuable mouth wash in cases of typhoid and other low forms of fever on account of its cleansing properties.

---

Dr. H. Fisher, St. Louis, Mo., believes that peroxide of hydrogen has a stimulating and healing influence upon inflamed tissue in addition to its cleansing, pus destroying properties. He thinks its therapeutic value in necrosed conditions is greatly increased by the free sulphuric acid it contains.

---

Dr. J. Allen Osman, Newark, N. J., says that in an experience of about fifteen years, he has never seen an amalgam filling that was perfect in its adaptation to the walls of the cavity after the lapse of two or three months except in very old, very black fillings, when the tooth was also discolored more or less, indicating copper as an ingredient of the amalgam; he says that the great secret in the use of copper amalgam is to get an extremely soft, pulpy mass; never attempt to use it in the dry, granular state. Smooth it off with wet bibulous paper; it will have a smooth, glassy appearance, and will not discolor unless it has been heated too much.

---

Dr. J. Allen further says, *don't* face copper amalgam with alloys of silver and tin—it won't work. On the other hand, a perfect result is gained by adding a gold finish after the amalgam has hardened, as both are non-shrinkable.

---

Dr. L Milliron, Kimball, D. T., advises the use of coffee without milk or sugar after the administration of chloroform, to aid in unloading the stomach of chloric acid, which forms there and causes headache. As a stimulent for anæmic patients, Dr. L. Milliron, Kimball, D. T., prescribes camph. tr., lavender camp., spts. sulph. ether, equal parts; dose, 15 to 30 drops as often as required.

---

Dr. M. Chas. Gottschaldt, New York City, suggests running the edge of sandpaper or corundum discs over a cake of dry toilet soap, giving a smooth edge which will glide over the rubber dam, preventing the catching which is so annoying.

---

Dr. J. C. Reese, Cameron, Texas, after two years' experience in the hypodermic injection of cocaine for the painless extraction of teeth, reports but one absolute failure and no alarming symptoms in any one case.

---

Dr. C. Swop, Boonville, Mo., works without an assistant, using short wooden-handled pluggers and a light wooden mallet, which, he claims, prevents all sense of jarring. In the handle of his mallet he has a needle set, which is cut through the eye to form a fork, with which he handles his foil.

---

Dr. D. I. McMillan, Kansas City, Mo., casts an all-gold crown, using a natural tooth for a model. He covers the tooth with a piece of rubber dam stretched smoothly and presses it into the soft side of a piece of cuttle fish. This he fastens with pins to a piece of soft charcoal, adjoining a depression in which his gold is melted, flowing directly into the cuttle fish mould through a V-shaped way. This gives a very smooth cast which needs no filing.

---

Dr. Dwight M. Clapp, Boston, Mass., uses a combination of amalgam and gold in large cavities in frail teeth. Using a matrix he fills the cavity with dry amalgam and packs gold upon the amalgam, using plastic or crystal gold in the beginning; the first pieces apparently disappear. This combination offers all the advantages of amalgam with the appearance and the hard surface of gold. He attributes the method to Dr. Norman Kingsley.

Dr. Jno. C. McCoy, Orange, Cal., after testing hydronapthol for two years, reports it a good disinfectant and antiseptic.

---

As a local application to the gums (in the removal of tartar, etc.,) Dr. W. O. Kulp, Davenport, Iowa, uses chloroform, oil of cloves, and tinc. aconite, equal parts, applied on cotton.

---

Dr. Geo. S. Staples, Sherman, Texas, reports in a lady patient, twenty-six years old, the two permanent canines present in place of the lateral incisors, with the temporary canines still firmly in place. Should she lose the temporary canines, and should the permanent laterals erupt, where will they probably appear?

---

Dr. A. H. Thompson, Topeka, Kan., says that minds vary as much as faces. Every man knows more about some particular thing than he does about others, and in this direction he has a peculiar faculty of looking at things different from that of every other observer. To this innate variability of mental conception are due the many controversies imposed upon a long-suffering profession and which make society proceedings and journals of the day a burden to the soul.

Dr. Thompson, in another place, says, extremes react upon each other, and check and guard against being led astray by fanatics and vagarists; the lines cross each other at the level of truth. He says, as we are but the epitome of the experience of our ancestors, we inherit their impressions with irregular distinctness, and the preponderance of one or another is among the accidents that stamp our individuality.

---

In a case of suppression of the salivary and buccal secretions of several months duration, reported by Dr. W. B. Haddon, London, Eng., perspiration had also notably diminished, and the lachrymal secretion was arrested. The tongue was devoid of epithelium, cracked in all directions and absolutely dry. The mucous membrane was smooth, shining and pale. The tonsils were natural and the salivary glands natural in size. The patient was benefitted by the use of jaborandi.

In catarrh of the antrum, Dr. Schiffers, Liege, Belgium, operates, with the aid of cocaine, through the foramen in the middle meatus of the nose, opening up a passage for the exit of the confined secretions by means of a curved probe-pointed bistoury. He thus avoids the extraction of a molar.

---

Dr. D. U. Crossman reports in the *Virginia Medical Monthly*, anomalous ducts of exit from parotid glands in six children in one family, four brothers and sisters and two cousins, the glands discharging continually through small orifices in front of the upper portion of the external ear, instead of pouring out through steno's ducts, their normal outlet. If these openings are not kept clean and clear, glandular irritation supervenes, and the secretion becomes very purulent, but it is very slight and scarcely noticeable when kept clean.

---

Dr. B. R. Stevens, Hannibal, Mo., fills root canals with blunt wood points dipped in chloro-percha and wrapped in warm very thin sheet gutta-percha, the latter projecting beyond the wood in a fine point. If the foraminal opening is very large the pulp-canal is moistened with creosote or carbolic acid and an impression taken by means of the above points, which serves as a guide for the preparation of a point for the permanent filling of the canal, which can thus be made very perfect.

---

As an exception to the rule that "teeth that naturally stand apart will remain sound," Dr. H. T. King, Fremont, Neb., found seven proximal cavities between the centrals and laterals, and right lateral and cupid, between which there was sufficient space to freely pass a No. 2 separating file. The other teeth, though in actual contact, were entirely free from proximal caries, with one exception in a lower first molar. The patient was a young lady of twenty years of age.

---

Dr. Chas. P. Grout, New York City, says that marble dust and glycerine 4 oz. glycerine to one quart marble dust, makes a beautiful cast, taking care not to have the metal too hot and not to pour it directly upon the palatine surface.

Dr. D. R. Stubblefield, Nashville, Tenn., emphasizes the point that the effect of cocaine travels both ways along the sensory nerve, towards the periphery as well as toward the brain; also that the effect is not cumulative.

---

In the use of cocaine for extractions, Dr. D. R. Stubblefield, Nashville, Tenn., obtunds the anterior dental nerves by means of the sup. max. branch of the fifth pair. Near its exit at the infra-orbital foramen, the inferior posterior teeth by the inf. max. branch, the posterior superior teeth by tapping the posterior dental nerves where they are given off in the spheno-maxillary fossa, the anterior inferior teeth by the mental nerves near the mental foramen. To reach the inferior dental nerve he enters through the cheek just posterior to the ramus.

---

Dr. R. R. Freeman, Nashville, Tenn., says that in every dental operation the object should be the best results, with the least pain, in the shortest length of time. If all these cannot be secured, seek a happy equilibrium, therein lies success.

---

Dr. W. W. H. Thackston, Farmville, Va., speaking of the influence of pregnancy upon the teeth says: "The nutrition and pabulum essential to the support and protection of dentine and enamel appears deficient in quantity and quality and is most likely diverted from its normal office by the exigencies and demands of foetal development, and as a consequence and result, the teeth suffer disintegration and decay.

---

Dr. H. E. Beach, Clarksville, Tenn., is of the opinion that more trouble has arisen from the neglect of needed operations on the teeth during pregnancy than ever did or ever will occur from careful, skillful attention.

---

Dr. M. L. Newborn, Memphis, Tenn., says of the ciliated epithelium of the antral cavity, the function of file after file and rank after rank of these little bodies is to sip its burden of mucous from its sister in the rear and kiss it away to the one in ro nt.

In chronic cases of purulency of the antrum, Dr. J. L. Newborn, Memphis, Tenn., believes that the mucous is secreted in a normal condition, but its natural means of exit being destroyed, by paralysis of the ciliated epithelium, it putrifies and infects the whole cavity, converting it into a pandora's box from which may originate neuralgia, vertigo, ulceration, necrosis, ozena, septicæmia, death.

In the treatment of chronic abscess of the antrum, Dr. J. L. Newborn, Memphis, Tenn., considers a drainage-tube absolutely necessary; this may be attached by clasps to the teeth, or to a plate, the pumping action of the tongue and mouth keeping the cavity free from mucous and purulent accumulations. If attached to a plate used in mastication, French rubber tubing may be used to avoid irritation, passing the tube through a hole drilled in the plate.

In the treatment of diseased antrum, Dr. J. L. Newborn, Memphis, Tenn., uses a common toilet perfume anatomizer. By heating the medicated wash to the right temperature, he fills the cavity with a fog of vapor, using iodine, carbolic acid and phenol sodique in varying proportions.

---

Dr. J. L. Newborn, Memphis, Tenn., reports two cases known to him, of chronic antral abscess terminating fatally, in from twelve to fourteen years, one with necrosis and pyæmia, the other with septicæmia; neither had proper treatment. He considers post-climative antral diseases in females positively incurable, recognizing in the systemic physiological changes attending that period a pre-disposing cause or factor in the production of purulency of the antrum.

Prof. W. L. Dudley, Vanderbilt University, concludes as the result of experiments upon the lower animals, that the toxic effects of cigarette-smoking are due to the inhalation of tobacco smoke, carbonic dioxide being its most poisonous constituent; that the smoke of a cigar or Turkish pipe would be equally as injurious if inhaled.

---

Dr. L. E. Custer, Dayton, Ohio, defines the histological structure of dentine as a tube enclosing a beaded thread of nerve tissue—the interbeaded spaces filled with a fluid supposed to be water—water being also a constituent of the fibril itself, and essential to the performance of nerve function. The water surrounding the fibril preserves an equilibrium of pressure and transmits sensation to the pulp. He says that ether spray as a local anæsthetic benumbs cutaneous tissue by the withdrawal of blood and nutrition from the parts; its action on the dentinal fibril is entirely dependant upon its refrigerant quality.

---

Dr. L. E. Custer, Dayton, Ohio, says it is not intended that a tooth should be refrigerated until icicles hang from it, nor that the pulp should be frozen in order to obtain success with the "Ottolengui method." He thinks that frequent repetition of the Ottolengui method would lead to a chronic lesion of the pulp and its destruction, but he sees no objection to a single, or a second operation.

Heroic Treatment.—"Heroism is what cuts through and burs out Atkinson's diseased apices of the roots; heroism is what surgically treats live pulps; heroism is what implants teeth.

---

Dr. H. A. Smith, Cincinnati, says that a tooth can not be subjected to great change of temperature without disturbance in the pulp; a pulp that has been subject to irritation would, by the repeated shocks of the Ottolengui method be most likely conveyed past the point where it could recover its physiological state.

---

Dr. J. Taft considers the Ottolengui method too energetic; that there is liability of producing periosteal irritation and of injuring the pulp to an extent that it would not recover on return of normal temperature.

---

Dr. J. R. Bell, Cleveland, Ohio, applies alcohol or peppermint on cotton to sensitive dentine followed by the hot blast two or three times in extreme cases.

Dr. F. S. Maxwell, Stenbenville, Ohio, distinguishes two forms of pericementitis: apical, or true pericementitis, caused by a dead or dying pulp, and augmented by the escape of gases through the apical foramen into the surrounding tissue; and gingival pericementitis, having its origin at the gingival border from causes, some of which are constitutional and chronic.

In calcic pericementitis with absorption of the alveolus, Dr. F. S. Maxwell, Steubenville, Ohio, uses a 30 per cent solution of chloride of zinc under the free margin of the gums.

In the after treatment of phagedemic pericementitis, Dr. F. S. Maxwell, Steubenville, Ohio, uses oil of cinnamon, two parts, to one part of carbolic acid, as a stimulant, to be used every three or four days, rinsing the mouth frequently with listerine slightly diluted.

If in the treatment of phagedenic pericementitis the gingival margin is left intact, Dr. F. S. Maxwell, Steubenville, Ohio, expects attachment between the cementum and the peridental membrane even of a dead tooth, with complete reformation of the peridental membrane; but just so far as the margin of the gum may have receded from its normal position, just so far will there be no reformation of the membrane.

---

Dr. J. J. McCulloch, New Orleans, says it is an illusion for any dentist to think he can get along without the knowledge gained through microscopical investigation; all the brilliant achievements of dentistry are due to the researches of the microscopist. He says that to insure a perfect amalgam filling the inside of the cavity must be much larger than the orifice, the edges nicely trimmed and polished, theamalgam rubbed briskly in a wedgewood mortar till a smooth mass is obtained which must be placed in the cavity piece by piece till the cavity is more than full, placing over it a tightly rolled ball of cotton and with a large ball burnisher pressing thewhole mass closely home. It should be finished two or threedays later with emory strips, pumice, and chalk. He suggests that the dentist who still practices pulp-capping is suffering from something more serious than an illusion; the record of the past proving it a failure. He is not an adherant of the germ theory. He says if any bugs remain alive after *one* thorough treatment of a root canal with carbolic acid, hot air, and chloroform, and immediate filling with an orangewood point and chloro-percha, the only harm they can possibly do is to eat the gutta-percha, and this they are welcome to do.

---

Dr. Bonchard, Paris Academy of Sciences, says that naphthol, as a disinfectant, is five times as active as carbolic acid, and three times as active as creosote. Corrosive sublimate is ten times as active as naphthol but it is also one hundred and eighty-seven times more poisonous. Naphthol is only slightly soluble and therefore is not absorbed, and so does not affect the general organism.

